# Wing Geometry Analysis of *Aedes aegypti* (Diptera, Culicidae), a Dengue Virus Vector, from Multiple Geographical Locations of Samut Songkhram, Thailand

**Published:** 2018-12-25

**Authors:** Tanawat Chaiphongpachara, Nattapon Juijayen, Kitthisak Khlaeo Chansukh

**Affiliations:** 1 College of Allied Health Sciences, Suan Sunandha Rajabhat University, Bangkok, Thailand; 2 Bachelor of Public Health, College of Allied Health Sciences, Suan Sunandha Rajabhat University, Bangkok, Thailand; 3 Department of Applied Thai Traditional Medicine, College of Allied Health Sciences, Suan Sunandha Rajabhat University, Bangkok, Thailand

**Keywords:** *Aedes aegypti*, Mosquito vector, Geometric morphometric, Dengue hemorrhagic fever

## Abstract

**Background:**

Dengue Haemorrhagic Fever (DHF) is a mosquito-borne disease and remains a major public health problem, especially in tropical and temperate countries. Studying wing morphometric of *Aedes aegypti* as a mosquito vector of DHF can help to better understand biological process of the mosquito adaptation to the environment. We aimed to study the geometric morphometric of *Ae. aegypti* from multiple geographical areas.

**Methods:**

Samples were collected from Samut Songkhram Province in Thailand, including coastal, residential and cultivated areas, by Ovitrap once per month during Oct to Nov 2016.

**Results:**

According to size variation analysis of *Ae. aegypti*, the female mosquito in a cultivated area was significantly different from those in the coastal and residential areas (P< 0.05). Whereas male *Ae. aegypti* in a cultivated area were significantly different from those in a residential area (P< 0.05). The shape variation of both female and male *Ae. aegypti* from all areas was statistically different (P< 0.05).

**Conclusion:**

Normally, living organisms, including mosquitoes, are adapted to their environment. The studied geographical locations affect *Ae. aegypti* morphology.

## Introduction

Dengue Haemorrhagic Fever (DHF) is a mosquito-borne disease and major public health problem in several countries worldwide (1). *Aedes aegypti* (Diptera, Culicidae) is the primary mosquito vector of DHF that carries and transmits dengue virus to humans. Moreover, *Ae. aegypti* is closely associated with humans (it is active in the daytime and prefers resting in and around human houses) (2). In addition, *Ae. aegypti* can be resistant to chemical insecticides (3). The combination of these factors is associated with outbreaks of DHF. The geographical environment affects the size and shape of mosquitoes (4). The geographical is relating to the landscape of the earth, where it has an environmental and habitat associated with mosquito vector (5).

Thailand is currently experiencing a disease epidemic. Regarding the current DHF situation in Thailand, the Bureau of Vector Borne Disease at the Department of Disease Control (within the Ministry of Public Health) has compiled retrospective data for recent years. Between 2011 and 2013, the DHF incidences rate were 100.789, 116.24, and 234.86 cases per 100000 population, the number of DHF patients is increasing year on year, and the disease remains a major public health problem in Thailand. Samut Songkhram is the smallest province in Thailand. Samut Songkhram is among those provinces with the most DHF cases (671 DHF patients [345.54 per 100000 population] in 2015). This province consists of a coastal area influenced by tides and having diverse coastal plants, a residential area (high population density), and a cultivated area (low population density). These environments produce morphological variation in *Ae. aegypti* (6). Studying the morphological variation of the mosquito helps us have a better understanding of causes, factors, and biological process of the mosquito’s adjustment to the environment (7). Recently, transmission of Dengue virus by *Ae. aegypti* in any geographic area depends on many factors, including extrinsic features related to the environment and intrinsic factors associated with the virus and vector interaction (6). Therefore, to reduce the presence of the vector, it is necessary to study the variability of mosquitoes as a basis for medical entomology.

Geometric morphometric (GM) is a newly developed morphometric technique for analysis of shapes and sizes of organisms using the principles of geometry (8). Many reports have applied GM to medical mosquitoes (9). GM can also be applied to study morphological variations of organisms and analyze the evolution of wing morphometric (10). As with *Aedes* spp., the GM technique was used to identify three species in Thailand (*Ae. aegypti*, *Ae. albopictus* and *Ae. scutellaris*), found to be highly capable (11). Molecular techniques are popular and powerful tools for the identification of species (12). PCR-based methods are often used for species identification because of their sensitivity, reliability, and specificity for species identification (13). In addition, molecular methods are also commonly used to study genetic variation across different areas (14). Although high-efficiency molecular techniques can use for species identification and genetic variation of mosquito, these are expensive and require specialized training (9). GM is an attractive alternative approach to identification and variation because it is cheap, easy, and fast.

We aimed to study the GM to investigate the impact of geography on *Ae. aegypti* from multiple geographical areas of Samut Songkhram Province, including coastal, residential and cultivated areas.

## Materials and Methods

### Mosquito collection

**Fig. 1.**
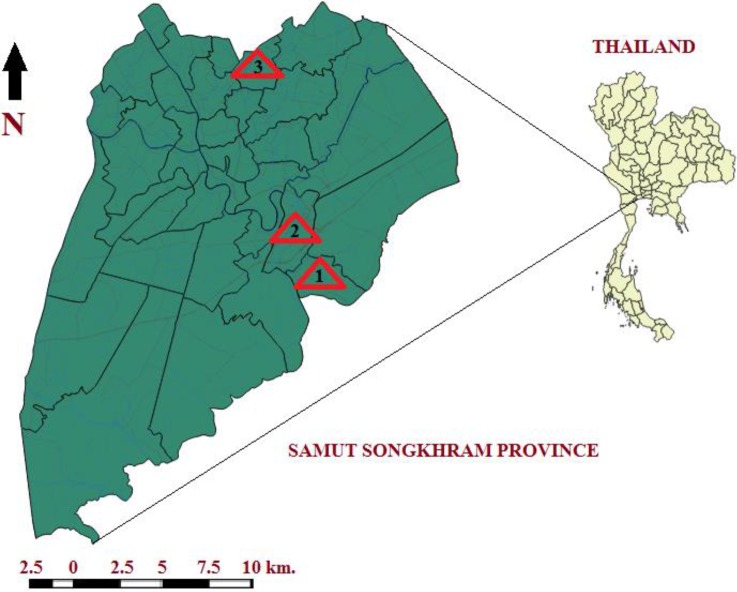
Map of *Aedes aegypti* collection sites in different geographical locations (1= Bang Cha Kreng Subdistrict as Coastal area, 2= Mae Klong Subdistrict as residential area, and 3= Jompluak Subdistrict as cultivated area)

Larvae of *Ae. aegypti* were collected in three different geographical locations across Samut Songkhram Province (coastal, residential, and cultivated sites) by ‘ovitrap’ once per month during Oct to Nov 2016. The geographical locations were selected by spatial data on land utilization information in Samut Songkhram from the Department of Provincial Administration of Thailand. The coastal area was Bang Cha Kreng Subdistrict (13° 23′31.57″N 100°1′59.36″E), the residential area was Mae Klong Subdistrict (13°24′32.52″ N 100°0′41.40″E), and the cultivated area was Jompluak Subdistrict (13°28′23.7″N 99°55′ 05.1″E) (Fig. 1). One village in each of the study areas reporting the highest dengue cases was selected, according to data from the report of Samut Songkhram provincial Bureau of Epidemiology in 2015. Six ovitraps (one trap per house) were set around houses or spaces under houses in each geographical location. Field collected larvae were then reared in the laboratory of College of Allied Health Sciences (Suan Sunandha Rajabhat University, Thailand). Emerged adults were morphologically identified by illustrated keys to the mosquitoes of Thailand (15).

### Mosquito preparation

Only the right wing of *Ae. aegypti* was analyzed. The right wings were dissected and mounted on microscope slides with coverslips using Hoyer solution. All of the sample wings were photographed using a Nikon DS-Ri1 SIGHT digital camera connected to a Nikon AZ 100M stereo-microscope (Nikon Corporation, Tokyo, Japan). The images were analyzed using the Morphometric CLIC Program.

### Geometric morphometric

**Fig. 2.**
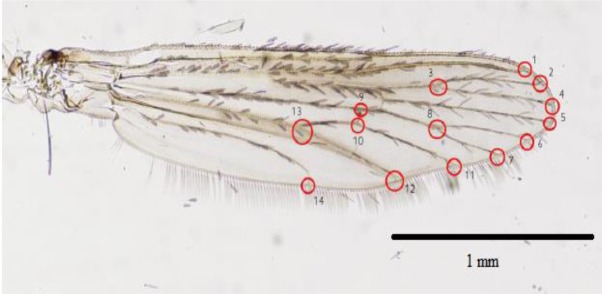
*Aedes aegypti* wing showing the 14 landmarks used in the morphometrics analysis

Fourteen landmarks were digitized (Fig. 2). For these selected landmarks, there is a selection criterion that must be clearly visible in order to prevent mistakes when plotting. The precision and measurement error of images was estimated by the ‘repeatability’(R) index (7, 8). Ten images of mosquito wing in each geographical location were randomly chosen for R testing and repeatability of plotting landmark from random images. After that, both two sets of images were computed as 1-R, with R, which is a Model II one-way ANOVA on repeated measures (7, 8). The landmark-based GM analyzed the size and shape of the mosquito. For wing size estimation, the size was measured by estimating the centroid size (CS) defined as the square root of the sum of the squared distances between the center of the configuration of landmarks and each individual landmark (11). Statistical differences between the centroid sizes of the male and female mosquito wings from the different areas were analyzed by non-parametric permutation tests (1000 cycles).

For wing shape evaluation, shape variables were measured and analyzed by principal components (PCs) of the “partial wrap” scores calculated after generalized Procrustes analysis of raw coordinates. Wing shape differences between geographical locations were calculated based on the Mahalanobis distance. Similar to wing size estimation, statistical differences in wing shape were analyzed by non-parametric permutation tests (1000 cycles). Neighbor-joining (NJ) trees were generated and calculated based on Procrustes distances between *Ae. aegypti* from different locations. Validated reclassification accuracies were estimated for testing variation of *Ae. aegypti* in each geography location yielded by GM, each individual was reclassified by comparing the shape based on the Mahalanobis distances.

### Software

Data collection and analyses were performed using the various modules of the CLIC version 97 (Collecting Landmarks for Identification and Characterization), which is freely available at http://xyom-clic.eu (7). The following modules used COO for landmark collection, TET for the transformation of data to be analyzed, MOG for centroid size and shape variables analysis to compute Procrustes distances, PAD for statistical significance analysis of shape variables and to compute Mahalanobis distances, and VAR for statistical significance analysis of size variables.

## Results

By applying the GM technique, we analyzed 220 samples comprised of 103 female and 117 male mosquitoes. According to geographical classification, 68 analyzed samples were from a coastal area, 82 samples from a residential area, and 70 samples from a cultivated area (Table 1). For repeatability, comparison of two sets of repeated measurements for the same images of *Ae. aegypti* wing for GM testing showed good scores (0.994).

**Table 1.** The number of *Aedes aegypti* used for analysis to classify by sex and geography

**Sex**	**Number of *Aedes aegypti***			

	**Total**	**Coastal Area**	**Residential Area**	**Cultivated Area**
**Female**	103	32	40	31
**Male**	117	36	42	39
**Total**	220	68	82	70

### Size variation

Size variation of *Aedes aegypti* wings was analyzed from the centroid size average of the wings from the different areas. Classified by sex, female mosquitoes in the residential area had the highest average (2.06vs 2.04 and 1.95mm) in the coastal and cultivated areas respectively. Similarly, male mosquitoes in the residential area had the highest average (1.62 vs 1.57 and 1.54mm) in coastal and cultivated areas respectively (Table 2). Female mosquitoes in the cultivated area had significant smaller wing size than those in the coastal and residential area (P< 0.05). Male mosquitoes from the cultivated area had smaller wing size than those from the residential area (P< 0.05) (Table 4).

**Table 2.** Means of wing centroid size of *Aedes aegypti* classified by sex and geography

**Sex**	**Geography**	**n**	**Means±SD (mm.)**	**Range (Min-Max)**
**Female**	Coastal Area	32	2.04±0.11	1.93–2.15
	Residential Area	40	2.06±0.21	1.85–2.27
	Cultivated Area	31	1.95±0.16	1.79–2.11
**Male**	Coastal Area	36	1.57±0.18	1.39–1.75
	Residential Area	42	1.62±0.15	1.47–1.77
	Cultivated Area	39	1.54±0.14	1.40–1.68

n= Number of Aedes aegypti

### Shape variation

**Fig. 3.**
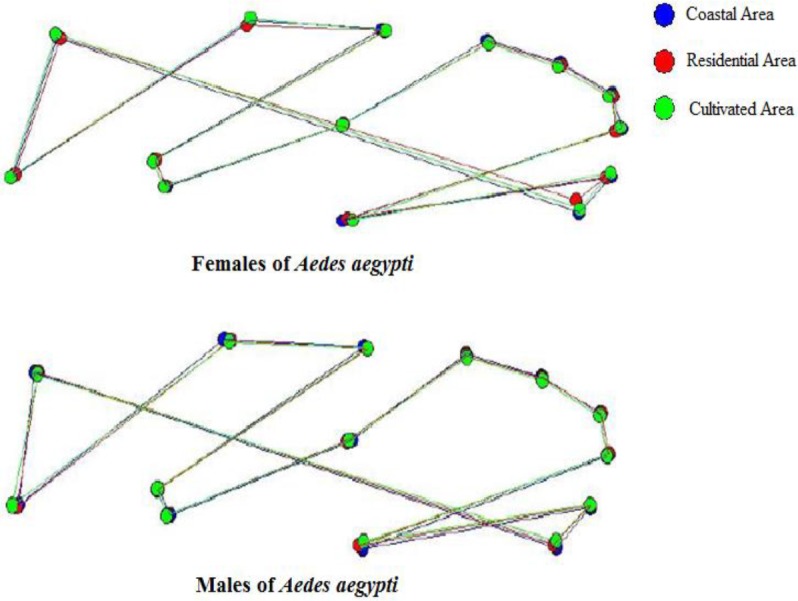
Superimposition of the mean landmark configurations of *Aedes aegypti* in different areas [Coastal area (blue), Residential area (red), Cultivated area (green)]. Top: females of *Ae. aegypti*, bottom: males of *Ae. aegypti*

**Fig. 4.**
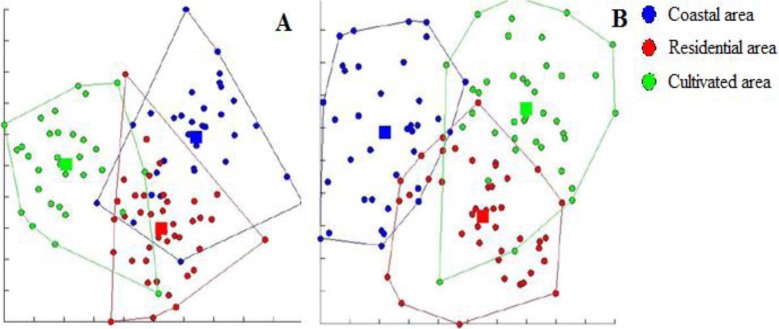
Factor map from landmark-based discriminant analysis by partial wrap for females (A) and males (B) of *Aedes aegypti* classified by geographical locations

After superimposition of the mean landmark configuration of the wings of the males and females from the different areas, polygons as connected mean landmark positions were demonstrated. Both polygons as connected mean landmark configurations of female and male mosquito in each different environmental types were clearly different (Fig. 3). Both factor map of female and male *Ae. aegypti* from landmark-based discriminant analysis by partial wrap showed overlapping of wing shapes in each different geographical type (Fig. 4).

**Fig. 5.**
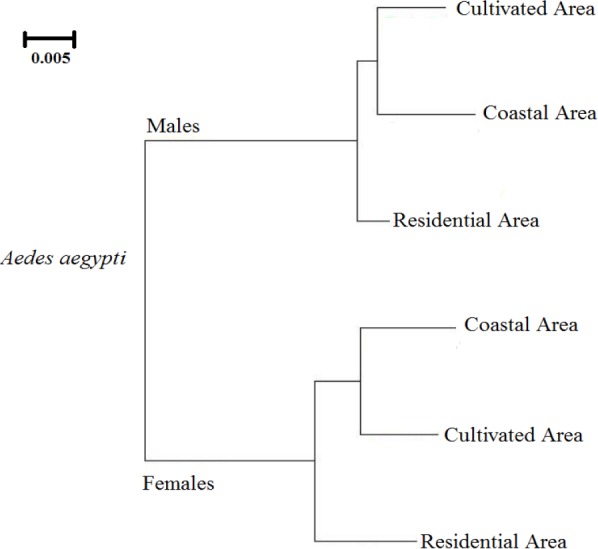
Neighbor-joining trees for shape based on GM analyses of male and female *Aedes aegypti* from different geographical locations

Mahalanobis distances of female *Ae. aegypti* wing shapes in cultivated and coastal areas had the highest value (3.80). Similarly, male *Ae. aegypti* wing shapes in the cultivated and coastal areas had the highest value (both 2.63) (Table 3). Tested by non-parametric permutation tests (1000 cycles), there was a statistical difference (P< 0.05) in both male and female of *Ae. aegypti* mosquitoes in each geographical location (Table 4). The neighbor-joining trees, based on the Mahalanobis distances between PCs, separated each sex and each location (Fig. 5).

**Table 3.** Mahalanobis distances between wing shapes of *Aedes aegypti* classified by sex and geography

**Geography**	**Females**			**Males**		
	
	**Coastal Area**	**Residential Area**	**Cultivated Area**	**Coastal Area**	**Residential Area**	**Cultivated Area**
**Coastal Area**	0.00			0.00		
**Residential Area**	2.53	0.00		2.06	0.00	
**Cultivated Area**	3.80	3.13	0.00	2.63	1.79	0.00

The validated reclassification accuracy scores confirmed separation from shape differences of *Ae. aegypti* in each geography location, males and females are slightly different. Reclassification scores of male mosquitoes were 80% to 89%, while these scores for female mosquitoes were 84% to 90%. The highest percentage of reclassification of male mosquito showed of 89% for the cultivated area, as well as the highest percentages of female mosquito was 90% of cultivated area. The lowest percentages of reclassification were 73% (male mosquito from the residential area) and 75% (female mosquito from the residential area) (Table 5).

**Table 4.** Statistical significance of size and shape differences of *Aedes aegypti* by non-parametric permutation tests (1000 cycles)

**Geography**	**Females**			**Males**		
	
	**Coastal Area**	**Residential Area**	**Cultivated Area**	**Coastal Area**	**Residential Area**	**Cultivated Area**
**Size**						
**Coastal Area**	0.00			0.00		
**Residential Area**	0.66	0.00		0.15	0.00	
**Cultivated Area**	0.01*	0.00*	0.00	0.35	0.02*	0.00

**Shape**						
**Coastal Area**	0.00			0.00		
**Residential Area**	0.00*	0.00		0.00*	0.00	
**Cultivated Area**	0.00*	0.04*	0.00	0.02*	0.00*	0.00

* Statistically significant (P< 0.05)

**Table 5.** Validated reclassification accuracies of male and female of *Ae. aegypti* in each geography location

**Geography**	**Percentage of reclassification**	

	**Male**	**Female**
**Coastal Area**	80% (29/36)	84% (27/32)
**Residential Area**	73% (31/42)	75% (30/40)
**Cultivated Area**	89% (35/39)	90% (28/31)

## Discussion

Here, GM was used to study the wing morphometric of *Ae. aegypti* in Samut Songkhram Province, Thailand. Two-hundred-twenty *Ae. aegypti* mosquitoes were collected from three different geographical sites.

### Size variation of mosquito

Females of *Ae. aegypti* have a larger centroid wing size than males, which is in line with other studies (10). Moreover, we detected a statistical difference in size variation of the female *Ae. aegypti*. The mosquitoes in the cultivated area were significantly different from those in the coastal and residential areas (P< 0.05). In addition, male *Ae. aegypti* were significantly different from those in the residential area (P< 0.05). The difference between *Ae. aegypti* in the cultivated area and the other areas might be a result of environmental factors, such as temperature, food quality and quantity, and suitable habitat (16). In fact, we did not study the environmental factors that affect *Ae. aegypti*. However, population density differences and the number of households in the cultivated area might have contributed to our findings. Other studies have addressed the relationships between population and household number and *Ae. aegypti* numbers (2). Urban areas are more suitable for *Ae. aegypti* than rural and sub-rural areas (17). Residential areas support the viability and breeding of *Ae. aegypti*, the mosquitoes can easily feed on human blood within houses, and there are many water containers (breeding sites for the DHF vector) (2). High nutrient levels produce larger mosquitoes (18). However, here we did not detect significant differences between the coastal and residential areas for either male or female *Ae. aegypti*. The might be because the coastal area of Samut Songkhram is a tourist attraction and, therefore, similar to the residential area (tourists plus water containers provide food and breeding sites).

Mosquitoes from the residential area were largest (female, 2.06±0.21, male, 1.62±0.15). In 2015, the Samut Songkhram Provincial Bureau of Epidemiology reported that this residential area produced more DHF patients than the other two tested areas. The body size of a female mosquito is correlated with fecundity, larger females lay more eggs during the first gonotrophic cycle (18). Moreover, male mosquito size correlates with total sperm numbers within a male and the number transferred to females (19). Thus, we would expect a relatively high density of DHF vector in the residential area, which is a factor of disease risk in areas of dengue virus transmission (20).

### Shape variation of mosquito

By visualized factor mapping of the landmark-based discriminant analysis (partial wrap), we detected separated and overlapping areas in the wing shape morphospace of male and female *Ae. aegypti* classified geographical location. After being tested with non-parametric permutation tests (1000 cycles), we found that the males and females wing shapes were statistically different in all geographical areas (P< 0.05), likely because of environmental factors (e.g., wind current and weather) (21). In each geographical location of Samut Songkhram Province, there were different environmental factors affecting *Ae. aegypti* shape, for example, storms and wind in the coastal area. The size and shape of *Ae. aegypti* and *Ae. albopictus* were examined from Nakhon Nayok Province of Thailand, Cucuta municipality of Colombia, and Florida and Hawaii in the United States and detected significant geographic differentiation (10).

Although we found no statistical difference in size variation in some areas, we detected shape variation across all groups. This might be because the mosquitos are adapted to their noticeably different environments. Geography has an impact on *Aedes* mosquito in each geographical location (22). Mosquito wing shape is correlated with mosquito population density and food quality. Studying these variations helps to better understand how *Ae. aegypti* adapts to its environment. In addition, such morphology data is important for taxonomy studies (11).

Samut Songkhram is a small province. Therefore, our ability to detect difference here suggests that GM is a very effective technique for studies addressing mosquito variation. Our validated reclassification accuracies scores demonstrate the efficiency of the separation in each geographical location. Thus, GM should be considered an alternative method for studying the variation of mosquito vectors.

## Conclusion

GM it is a newly-developed morphometric technique used to classify medical insect types and study morphology variants (8). GM is advantageous because it is easy to use, is low-cost, and rapid. GM does not require high entomological skills (7). GM is an effective tool for studying morphology variants of *Ae. aegypti*. *Aedes aegypti* from different locations differ in size and shape, which is important for understanding its local adaptation. Such knowledge might help to control mosquitoes in DHF endemic areas.
